# Wind Speed during Migration Influences the Survival, Timing of Breeding, and Productivity of a Neotropical Migrant, *Setophaga petechia*

**DOI:** 10.1371/journal.pone.0097152

**Published:** 2014-05-14

**Authors:** Anna Drake, Christine A. Rock, Sam P. Quinlan, Michaela Martin, David J. Green

**Affiliations:** Center for Wildlife Ecology, Department of Biological Sciences, Simon Fraser University, Burnaby, British Columbia, Canada; University of Milan, Italy

## Abstract

Over the course of the annual cycle, migratory bird populations can be impacted by environmental conditions in regions separated by thousands of kilometers. We examine how climatic conditions during discrete periods of the annual cycle influence the demography of a nearctic-neotropical migrant population of yellow warblers (*Setophaga petechia*), that breed in western Canada and overwinter in Mexico. We demonstrate that wind conditions during spring migration are the best predictor of apparent annual adult survival, male arrival date, female clutch initiation date and, via these timing effects, annual productivity. We find little evidence that conditions during the wintering period influence breeding phenology and apparent annual survival. Our study emphasizes the importance of climatic conditions experienced by migrants during the migratory period and indicates that geography may play a role in which period most strongly impacts migrant populations.

## Introduction

Every year, it is estimated that six billion songbirds leave wintering regions in Africa and Central and South America and redistribute themselves across Europe and North America [1]. These migratory species, which pollenate plants, disperse seeds, or consume insects and small mammals, quadruple bird abundance in the north and play a critical role in northern ecosystems [2]. Population declines among many migratory songbirds have led to a renewed interest in when and where factors influence their demography [3]–[5]. However, the geographic scale of migratory movement and our limited knowledge of the linkage between wintering and breeding populations, pose a challenge for ecologists and conservation biologists [5].

Climatic conditions are capable of impacting the population dynamics of migratory songbirds at all stages of the annual cycle. Conditions during the breeding period can alter migrant productivity by impacting total food availability and the timing of breeding events (e.g. [6], [7]). Within wintering regions, climate can directly influence songbird survival [1] and, via shifts in the timing of spring migration, indirectly influence productivity [8]–[11]. Conditions experienced during migration can influence stopover decisions and survival [4], [12], [13], although fewer studies exist and this period remains the most difficult to examine [5]. Understanding the relative importance of conditions at each stage of the annual cycle in explaining changes in population numbers becomes increasingly important as climate change is expected to impact regions differently [14].

In this study, we evaluate the relative importance of conditions during the winter, spring migratory, and breeding season in influencing the survival and breeding phenology of yellow warblers in western Canada, explicitly recognizing that events at one period may impact processes occurring in periods that follow. We developed climate models that considered the impact of: (1) rainfall patterns on wintering grounds, (2) wind speed and rainfall on the migration route in spring, and (3) temperature conditions on the breeding grounds in May. Rainfall in overwintering areas, through its influence on primary productivity and insect abundance, was expected to increase survival and allow birds to migrate earlier [15], [16]. Strong winds and precipitation impact the energetic cost and speed of movement during migration, and hostile conditions were expected to reduce survival and delay arrival [12]. Finally, local temperatures at the beginning of the breeding season impact the timing of insect development and therefore food availability [17] and migratory birds may adjust arrival and/or clutch initiation in response to these local/regional conditions [6].

## Materials and Methods

### Ethics statement

Targeted mist-netting and banding of individuals was carried out in accordance with Canadian Council on Animal Care recommendations and under permits issued by Environment Canada (CWS Banding Permit: 10759H; Scientific Permits: BC-SCI-59-04-0335; 59-05-0328; 59-06-0347; 59-07-0331; 59-08-0388; and BC-09-0296; 10-0022; 11-0037; 12-0010). Field protocols were approved by the University Animal Care Committee at Simon Fraser University (Protocol # 709B-04, 869B-04, and 1038B-04).

### Study species

Yellow warblers (*Setophaga petechia*) are small, insectivorous neotropical migrants with a broad breeding distribution in North America. Genetic-isotopic work indicates eastern and western lineages have parallel migration systems and differing wintering distributions [18]. Birds that breed in the northwestern region of the continent winter primarily in lowland areas within the occidental and isthmus region of Mexico [18] where they occupy coastal scrub, riparian corridors, agricultural habitat, second growth, and tropical evergreen forest [19], [20]. Migratory yellow warblers are present on the western and central flyways between March and May [21].

### Study system

We have studied a population of yellow warblers breeding in Revelstoke, British Columbia (50.97°N, -118.20°W) for nine years (2004-2012). Colour-banded and monitored individuals breed within three, 30-39 ha plots of seasonally flooded grassland interspersed with isolated willow thickets (*Salix* sp.) and black cottonwood *(Populus trichocarpa)* forest along the northern section of the Upper Arrow Lakes Reservoir (elevation 435-441 m). All banded individuals are aged as either “young” (SY; first breeding season) or “older” (ASY; at least 2 years old) birds based on plumage characteristics [22].

### Survival and breeding phenology

Our 2004-2012 mark-resight dataset contained 279 individuals (141 females, 138 males) and 460 annual encounters. Detailed breeding data was collected from 2005-2006 and 2008-2012. In these years, the study plots were surveyed every 1-2 days from early-May to late-June to determine male arrival dates. Breeding pairs were then monitored every 3 days until late-July in order to determine when females initiated their first clutch, and document the fate of all nesting attempts. Nestlings were banded seven days post-hatch and the number of nestlings present at this date was assumed to be the number of young fledged from nests where fledging was subsequently confirmed and evidence of predation was absent. Annual productivity was defined as the total number of young fledged across all nesting attempts made by a given individual (for further detail see [20]).

### Climate models

#### Climate conditions on the wintering grounds

We used standardized monthly Southern Oscillation Index (SOI) values [23] to describe climatic conditions on the wintering grounds because the El Niño Southern Oscillation (ENSO) impacts rainfall patterns in Mexico [24]. Within the occidental and isthmus regions of Mexico, the majority of rainfall (>60%; [24]) occurs during the summer monsoon (May-August). Weak monsoon years are associated with El Niño phases of ENSO [24] and likely result in reduced food availability for birds wintering in this region. We therefore predicted that negative mean SOI values (dry, El Niño conditions) in the May-August period would be associated with low survivorship and delayed phenology in our population the following spring (Model 1: SOI_MAY-AUG_). It is possible that late winter rainfall is disproportionately important to neotropical migrants. Such rainfall contributes significantly less moisture to over-winter habitat than the monsoon but occurs at a critical time, when regions are experiencing drought conditions. In winter El Niño conditions (negative SOI) promote greater precipitation [24]. We therefore predicted that negative SOI values in the December-March period would be associated with favorable conditions that would improve survivorship and advance breeding phenology (Model 2: SOI_DEC-MAR_).

#### Climate conditions on spring migration

**Figure 1:**
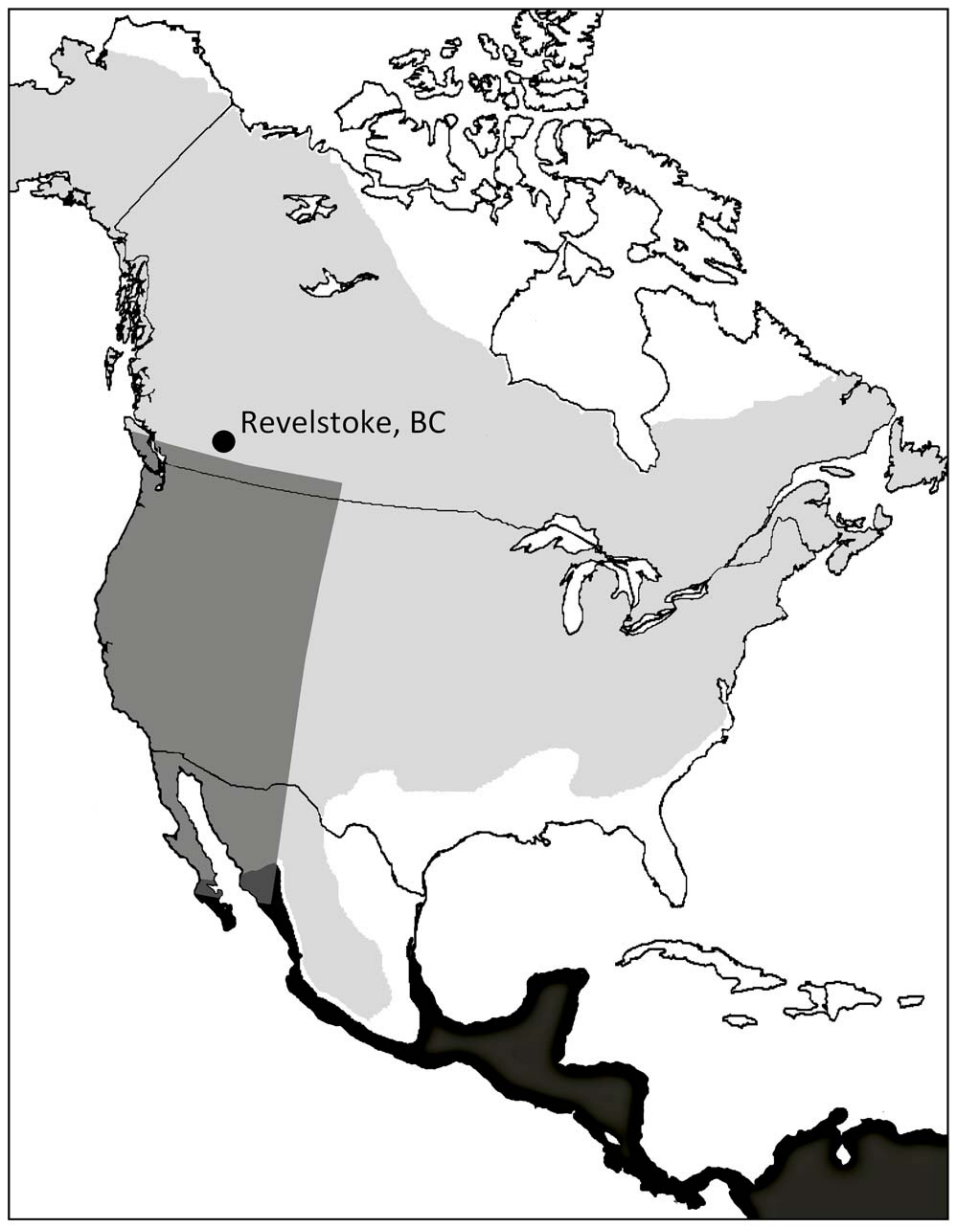
The location of our study, the yellow warbler breeding range (light grey) and wintering range (black), and the area used to calculate wind speed values during migration (dark grey). Base map: Wikimedia Commons.

Conditions experienced during spring migration could alter both timing and survival rates. We used mean nighttime wind vectors (westerly (U-) and southerly (V-) components (m/s)) and precipitation (Kg/m^2^/s) between March and May as measures of migration costs. Variables were derived from modeled climate data extracted from the National Center of Environmental Prediction (NCEP) Reanalysis 1 data archives at the NOAA-CIRES Climate Diagnostics Center at Boulder, Colorado, USA [25] using the RNCEP program [26]. These data have a spatial resolution of 2.5° latitude and longitude and temporal resolution of six hours. We defined the western flyway for our population as the overland region west of the easternmost portion of the continental divide (107°W), beginning at the northern extent of the yellow warbler wintering range (25°N) and ending at the latitude of our study site (50°N) (figure 1). U- and V-wind speed components were averaged from the 850 mb (∼1500 m AMSL) and 925 mb (∼700 m AMSL) level, and thereby encompassed conditions within much of the altitudinal range of migrant songbirds (e.g. [27], [28]). Rain and wind vectors were then averaged for the March-May period, dropping noon values so that the series represented conditions encountered during nighttime migration (between 18:00h and 6:00h). Within our defined migratory region during the March-May period, averaged U-winds were westerly in all years of our study and V-winds were southerly in all years except 2008. We expected high wind speeds (westerly (Model 3: U-wind), southerly (Model 4: V-wind), and combined (Model 5: U-wind + V-wind)) and greater precipitation (Model 6: Migration Rain) to be associated with reduced survivorship and delayed breeding phenology.

#### Climate conditions during breeding

We used averaged daily mean temperatures (°C) at our study site during the month of May to parameterize breeding conditions. This metric has been shown to be correlated with when yellow warblers initiate breeding events, likely due to its influence on prey availability [6]. Warmer springs may also result in greater food availability and earlier fledge dates, affording breeding pairs additional energy and time to moult and fatten for fall migration. Temperatures were drawn from “Revelstoke A” weather station (ID: 1176749; 50°57′40N, -118°11′00W, elevation 444.7 m [29]). Higher mean May temperatures at our study site were expected to be associated with earlier breeding phenology in the same year, and improved adult survivorship in the following year (Model 7: May°C).

### Analysis

We estimated apparent annual adult survival for the period 2004-2012 using the Cormack-Jolly-Seber model. We calculated the probability of an adult returning to the study site (φ) after adjusting for the re-sighting probability of banded individuals (*p*) using program MARK (5.1) [30], [31]. Probability-of-return (φ) reflects both survival and emigration, thus our apparent annual survival estimates do not include surviving individuals who permanently emigrate from the study site. The global model that allowed adult survival to vary as a function of gender, age and year, and re-sighting probability to vary as a function of gender and years where detailed breeding data was collected (2005–2006 and 2008–2012 vs. 2007), fit the data well (median procedure, ĉ = 1.09).

We first determined the best model structure for the re-sighting rate, and then modeled survival rates with candidate models containing gender, age, year, and all possible interactions. We used Akaike's Information Criterion corrected for small sample sizes and over-dispersion (QAIC*_c_*), to rank competing models. Re-sighting rates were best modeled with only a gender term; males were estimated to have higher re-sighting probabilities than females (male  = 0.92±0.04, female  = 0.65±0.08). Apparent annual survival rates were best described by a model that included year and age (AIC weight  = 0.53) or a model that included year, age and gender (AIC weight  = 0.41). The influence of climate variables were therefore investigated using a candidate model set that included models with: (1) only age and climate variables as main effects, (2) age, gender, and climate variables as main effects, and (3) main effects, age×climate interactions and/or gender×climate interactions (n = 47 individual models, table S1). Models were ranked using QAICc [31].

To assess regional climate effects on male arrival and female clutch initiation, we created a candidate model set that included linear models with age and climate variables as main effects, and linear models that included ‘age×climate’ interactions (n = 15 individual models, table S2). Models were run in R version 2.14.1 (2011, The R Foundation for Statistical Computing) and ranked using AICc. We subsequently created a data subset restricted to older individuals (2+ years) that returned to the study site in more than one year (males, n = 58; females, n = 26). We used this dataset to investigate whether climate variables that influenced breeding phenology at the population level also explained year-to-year variation in the breeding phenology of individuals.

Three of the climate variables we examined exhibited a temporal trend (table S3). We report our analysis of non-de-trended climate variables here [32] because we were interested in how absolute variation in climate influences population parameters, not in how year-to-year deviation from short-term trends account for unexplained variance in our population parameters. De-trended survival analyses had greater model uncertainty (table S4), but de-trended breeding phenology analyses yielded similar results to those obtained using non-de-trended climate variables (table S5).

We used path analysis to estimate how breeding delays associated with climate conditions influenced the annual productivity of young and older females [33]. For each age-class we first calculated the total effect (TE) as the product of the two path coefficients (standardized partial regression coefficients (β)) in the pathway between climate, clutch initiation date, and annual productivity. We then used the TE score, the standard deviation of the climate variable, and the standard deviation in the age-specific annual productivity of females in our population to predict annual productivity across the observed range of climatic conditions [33].

We report the relationship between mean SOI values (March-May; [23]) and wind speed (March-May) over a 50-year period (1963–2012) to allow comparison with other studies that have examined El Niño climate cycle effects on migrant survival and breeding phenology. We additionally use this dataset to assess patterns in wind speed over the March to May period.

## Results

### Survival and breeding phenology

**Figure 2:**
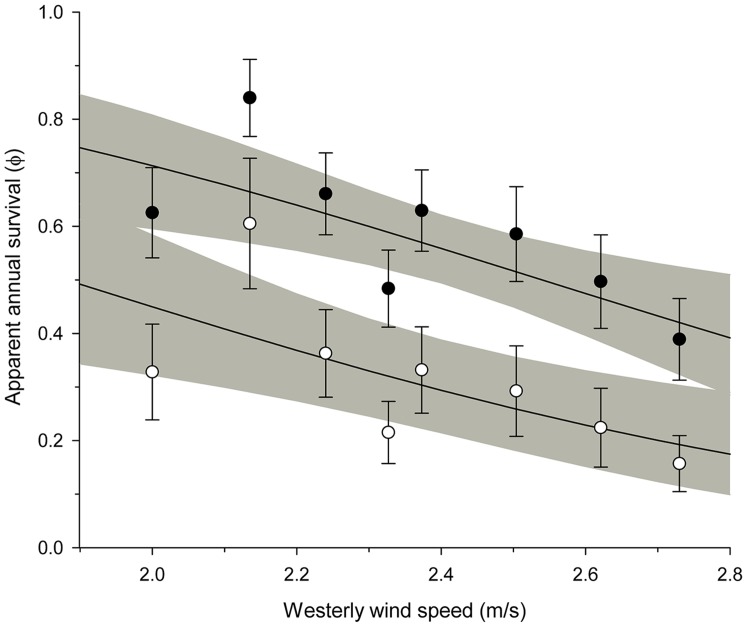
Relationship between westerly wind speed during migration and apparent annual survival of yellow warblers. Points are apparent annual survival (± SE) of young (1 yr, open points) and older (≥2 yrs, filled points) birds for 2004 to 2012. Solid lines and shading represent predicted apparent annual survival (φ) ±95% CI from the top model assuming an average southerly wind vector.

Inter-annual variation in the apparent annual adult survival of yellow warblers breeding in Revelstoke was best described by a model including migration wind speed (table 1). Wind speed models (3, 4, 5) received 79% of the total model support (table S1). Wintering models (1, 2) and the breeding period model (7) received 13% and 3% of the total model support, respectively. The top model (3a) indicated that apparent annual survival varied with age and declined as westerly wind speeds increased (figure 2).

**Table 1:** Climate models obtaining substantial support (ΔQAICc ≤2) and the base temporal model (‘Year + Age’) describing apparent annual survival of yellow warblers breeding in Revelstoke, British Columbia (n = 279 individuals, 460 encounters).

Model #	Period	Variables	K	QAICc	ΔQAICc	ω_i_
3a	Migration	U-WIND + AGE	5	613.61	0	0.197
5a	Migration	U-WIND + V-WIND + AGE	6	614.28	0.67	0.141
3b	Migration	U-WIND + AGE + SEX	6	615.08	1.47	0.094
5e	Migration	U-WIND + V-WIND + AGE + SEX + U-WIND*SEX + V-WIND*SEX	9	615.33	1.72	0.083
3c	Migration	U-WIND + AGE + U-WIND*AGE	6	615.52	1.91	0.076
-	-	YEAR + AGE	11	616.94	3.33	0.037

Model numbers match those described in the text. Age was included in all models as a covariate (*see* Methods).

**Figure 3:**
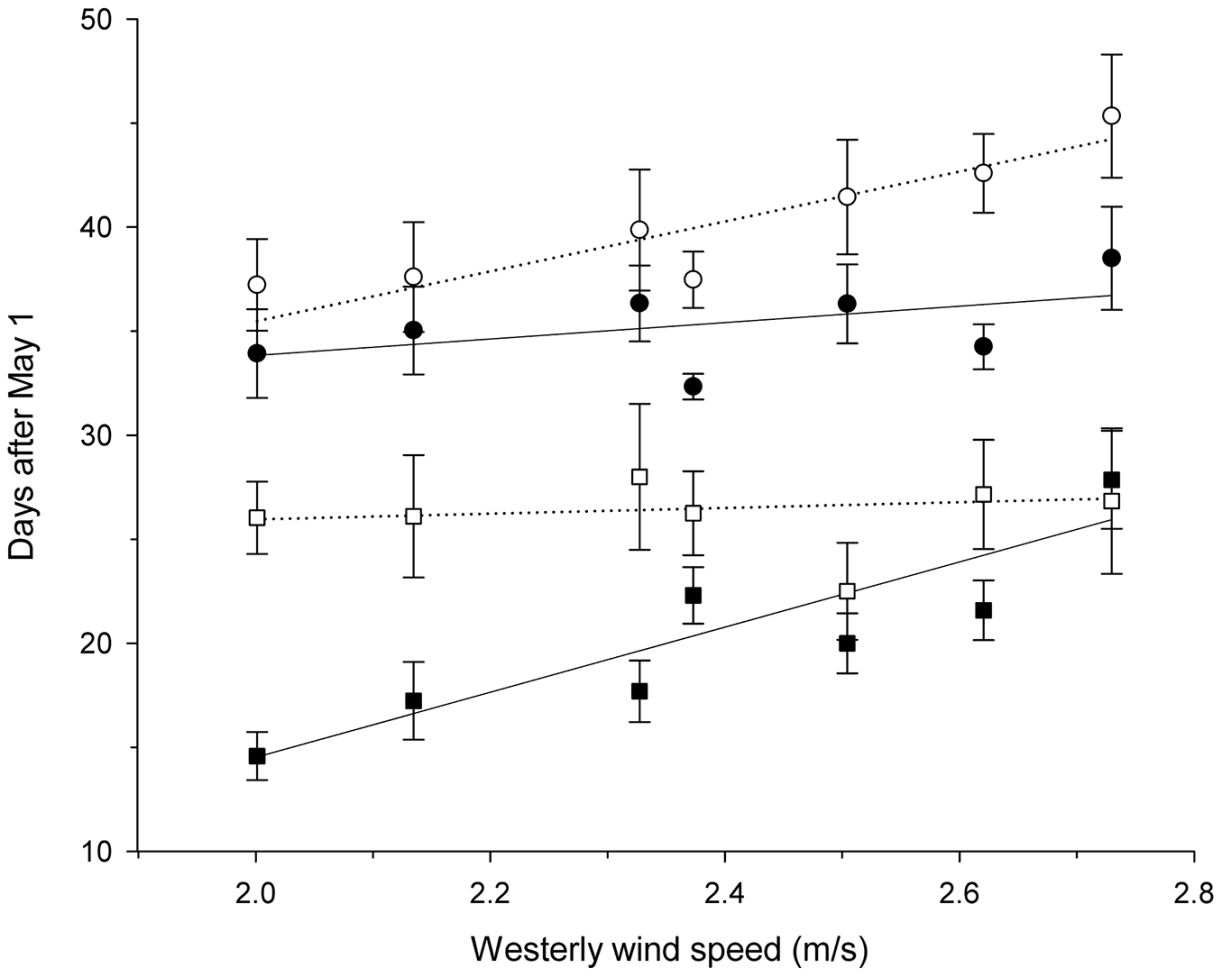
Male arrival date (squares) and female clutch initiation date (circles) for yellow warblers as a function of westerly wind speed during migration. Points represent mean dates ± SE for young (1 yr, open points) and older (≥2 yrs, filled points) birds in 2005–2006 and 2008–2012.

Annual variation in male arrival and female clutch initiation dates was also best described by models including migration wind speed (table 2). Wind speed models describing male arrival date received 66% of the total model support and wind speed models describing female clutch initiation date received 88% of total model support (table S2). The top model in both candidate model sets was (3b) which included westerly wind speed, age and a ‘wind speed×age’ interaction (table 2). The arrival of older males was delayed as westerly wind speed on migration increased, whereas the arrival of young males – who arrived later than older males – did not vary with wind speed (figure 3). Females of both age classes were delayed in initiating their first clutch in years with stronger westerly winds on migration but older females were less sensitive to variation in wind speed than younger females (figure 3).

**Table 2:** Climate models obtaining substantial support (ΔAICc≤2) and the base temporal model (‘Year + Age’) describing male arrival date (n = 210) and female clutch initiation dates (n = 177) for yellow warblers in Revelstoke, British Columbia.

Model #	Period	Variables	r^2^	K	AIC.c	ΔAICc	ω_i_
Male Arrival:							
3b	Migration	U-WIND + AGE + U-WIND*AGE	0.21	5	1452.88	0	0.535
1b	Winter	SOI_MAY-AUG_ + AGE + SOI_MAY-AUG_ *AGE	0.20	5	1453.85	0.98	0.328
-	-	YEAR+AGE	0.18	9	1464.32	11.44	0.002
Female clutch initiation date:							
3b	Migration	U-WIND + AGE + U-WIND*AGE	0.16	5	1194.89	0	0.289
5a	Migration	U-WIND + V-WIND + AGE	0.15	5	1195.00	0.11	0.273
3a	Migration	U-WIND + AGE	0.14	4	1195.82	0.93	0.181
5b	Migration	U-WIND + V-WIND + AGE + U-WIND*AGE +V-WIND*AGE	0.16	7	1196.47	1.58	0.131
-	-	YEAR + AGE	0.16	9	1197.90	3.00	0.064

Age was included in all models as a covariate (*see* Methods). Wintering model support for male arrival date was counter to prediction (*see* Discussion).

**Figure 4:**
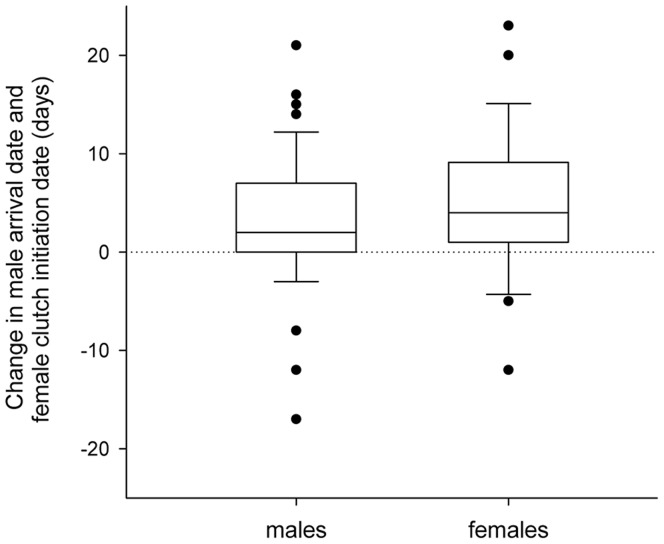
Within-individual changes in breeding phenology (male n = 58, female n = 26) associated with inter-annual variation in average westerly wind vectors. Box-plots show the median change, 10^th^ and 90^th^ percentile, and outliers. Positive values represent delayed breeding phenology in years with stronger winds on migration. Data was restricted to individuals that were ≥2 years of age in both years.

Differences in the arrival date of individual males and the clutch initiation date of individual females were also associated with inter-annual variation in westerly wind speed. Individual males exhibited a median delay of 2 days (t_57_ = 3.66, P<0.001) and individual females exhibited a median delay of 4 days (t_25_ = 3.22, P = 0.002) in years where westerly winds were stronger (figure 4).

### Carry-over Effect on Female Productivity

Annual productivity declined with later clutch initiation dates (older females: F_(1,103)_ = 8.25, P = 0.005, β = −0.27; young females: F_(1,68)_ =  14.51, P<0.001, β = −0.42). The total effect of westerly wind speed on annual productivity was consequently −0.03 for older females and −0.16 for young females. Based on these scores, older females would be expected to produce 0.2 fewer fledglings and younger females would be expected to produce 0.8 fewer fledglings in our highest versus lowest wind speed years.

### Wind speed variation and ENSO

Within the western flyway region (figure 1), average westerly wind speeds and SOI between March and May were correlated positively over the 50-year period we examined (r = 0.61, n = 50; Spearman's ρ = 0.55; P<0.0001). Thus, stronger westerlies on the Pacific side of the Rocky Mountains are associated with La Niña conditions in the central Pacific. We did not observe a relationship between southerly wind speeds and SOI within the same period and region (r = −0.12, n = 50, Spearman's ρ = −0.17; P = 0.24).

All wind speed vectors declined from March to May (westerly wind: F_(2,147)_ = 3.38, P = 0.04; southerly wind: F_(2,147)_ = 14.04, P<0.001). Westerly wind speeds were lower in May than in March and April while southerly winds showed a steady decline over the three-month period.

## Discussion

Research conducted on neotropical migrants that over-winter in the Caribbean and breed in eastern North America and on palaearctic-African migrants indicate that populations are limited by climatic conditions during the wintering and/or breeding period [1], [7], [34], [35]. In contrast, within our western North American yellow warbler population we find only weak evidence that climatic conditions during the wintering and breeding periods influence survival and phenology. Our findings strongly support the argument that avian populations can be limited during the migratory period [4]. Not only did conditions on migration best describe variation in annual survival within our population but these conditions also best described the timing of breeding, which in turn strongly predicts annual productivity within our population. To our knowledge, this is the first study to demonstrate that conditions experienced by birds on migration can impact both survival and productivity.

Migration is known to be a period of high mortality for migratory birds [36] and climate conditions on migration can theoretically limit migrant numbers [4], [37]. Higher westerly wind speeds during migration may be associated with lower apparent annual survival in our study because they reflect storm events that increase the risk of in-flight mortality [4]. High wind speeds that oppose the direction of spring movement should also increase flight costs and refueling requirements, and delay departure from stopover sites [12]. Resulting increases in migrant density, feeding effort, and competition at stopover sites may consequently increase the risk of starvation and predation *en route* [4]. However, declines in apparent annual survival may not necessarily indicate mortality. Changes in this metric associated with shifts in wind speed could also be a product of individuals being blown off course or “dropping out” of migration earlier in years when migration conditions are hostile. These individuals would then breed further east or further south and not return to our study site in subsequent years. As younger birds are less likely to have bred successfully at our study location, they may be more willing to forgo the benefits of philopatry [38], [39]. If climate effects on apparent annual survival are a product of dropping out, then we might expect young individuals to show a stronger response to wind speed. However, we found relatively little support for survival models that included a ‘wind speed×age’ interaction term (*see also* figure 2).

Increased wind speed on migration was associated with later male arrival dates and later female clutch initiation dates. This relationship was also supported by shifts in the phenology of individuals who returned to our study site in multiple years. The effect of wind speed on breeding phenology varied with age in both sexes. Older males arrived at our study site 12 days before younger males when wind conditions on migration were favorable and at the same time as young males when conditions were more hostile (figure 3). Given that wind speeds are lower later in the migratory period, adult male arrival dates may reflect a trade-off between the advantages of early arrival and the costs of early migration. Consistently “late” arrival dates among young males may reflect cost-minimization alone. In contrast to males, older females appeared to be less sensitive to variation in wind speed than young females (exhibiting a 3 day vs. 9 day delay in clutch initiation dates across the observed range of wind speeds). Older females may be better than young females at compensating for arrival delays if experience allows them to pair and initiate breeding more rapidly.

Conditions on migration that delay reproduction can decrease productivity because later clutch initiation dates are associated with reduced fledging success [40]. We found that wind-induced delays in breeding phenology in our population could reduce the annual productivity of young and older females by as much as 0.8 and 0.2 fledglings, respectively. These declines are significant as yellow warblers typically only raise a single brood of three – five nestlings and average productivity in our population is low (2.2±1.9 fledglings per year [38]).

We found no consistent evidence that wintering climate impacted our population. In males, the mean SOI index that predicts monsoon rainfall (SOI_MAY-AUG_) was associated with the timing of arrival on the breeding grounds. However, counter to our *a priori* prediction, this model indicated that males arrived earlier in years with a more negative SOI_ MAY-AUG_ index, when there is less monsoon rain and conditions on wintering territories should be worse than in years with a positive SOI_ MAY-AUG_ index (e.g. [41]). Food availability immediately prior to migration could be higher in years with less monsoon rain if aquatic insects emerge as ephemeral water-bodies disappear [42]. This might allow males to leave earlier. However, in the climate zone typical of the occidental and isthmus region of Mexico, moist habitats make up a small percentage of landscape and water-limitation is more likely to reduce food availability for yellow warblers [16]. We therefore believe that support for this model instead reflects the positive correlation between SOI_MAY-AUG_ and westerly wind speed on migration (U-wind) (table S3).

Previous work on the western flyway by Macmynowski et al. [43] and Nott et al. [44] indicate that the movement of migrants through California is delayed in La Niña years while migrant productivity in the Pacific Northwest is higher in El Niño years. These patterns were attributed to increased spring rainfall in the northern region of the Pacific slope of Mexico as well as more favorable southerly winds between March and May in El Niño years [44]. However, our analysis of ENSO and wind vectors within the “western flyway” (including California and the Pacific Northwest; figure 1) indicate ENSO is poorly correlated with southerly winds, but that La Niña conditions (+ve SOI values) are associated with strong westerly winds blowing off the Pacific during migration, while El Niño conditions (-ve SOI values) are associated with weaker westerlies. Our findings therefore corroborate those of Macmynowski et al. [43] and Nott et al. [44] but implicate westerlies as a possible additional explanation for their observations.

For our yellow warbler population, apparent annual survival models that included pre-migration rain (SOI_DEC-MAR_) or migration rainfall received little support (table S1). In contrast, apparent annual survival of Swainson's thrush (*Catharus ustulatus*) in the northwestern United States is positively correlated with higher spring rainfall in Sinaloa, Sonora and southwestern California [45]. Lack of support for migration rain in our study may be due to differences in the period and region over which our rainfall values were calculated. Rainfall in the southwestern portion of our migratory region during the pre-migratory period may be important with respect to food availability *en route* [44], [45]. As higher spring rainfall in this region is associated with El Niño and therefore reduced wind speed years, further work is needed to assess the independent effect of each variable on migrants.

The majority of studies conducted in western Europe and eastern North America that have examined the impact winter conditions on migrant songbird survival or reproduction have found support for wintering effects (e.g. [34], [35], [46] *but see* [7]). In contrast, only three western and central North American studies have found evidence of wintering conditions influencing breeding populations [11], [20], [44] while four have found either no support for wintering effects, or greater support for non-wintering effects ([6], [34], [45], this study). Migrant populations that breed in western Europe and eastern North America encounter large ecological barriers during migration (the Mediterranean sea, Sahara desert, the Gulf of Mexico and the Caribbean sea). Songbirds using migratory routes in western North America only cross the smaller deserts of the American southwest and those using central routes may avoid barriers altogether. Such geographical differences may be important in determining when in the annual cycle migrants are most impacted by climatic conditions. In regions where birds must cross large barriers, population responses linked to overwintering conditions may instead be the product of conditions encountered at pre-barrier stopover sites as these sites often overlap with wintering areas (for example, areas encompassed in [4], [8], [11]). Populations that are not required to make long, uninterrupted flights may compensate for poor departure conditions by shortening flight distance and increasing stopover number [47]; weaker support for wintering effects among these populations, including our study population, may be the result of a weaker dependency on any one wintering or stopover region to fuel migration.

Pacific wind patterns may explain the strong relationship between migratory conditions and demography in our study. These winds oppose the north/northwesterly course of spring migration and therefore represent a climatological barrier to spring movement. In contrast, winds encountered in central and eastern regions of the USA facilitate the northeasterly movement of migratory birds in those regions [48]. We suggest that, whereas pre-migration and, potentially, pre-barrier fattening in wintering regions may be essential for the movement and survival migrant populations in western Europe and eastern North America, *en route* conditions and habitats are more important for migrants in western North America (*see also* [45]). Our findings indicate that migration is the most costly period of the annual cycle for western neotropical migrants, impacting both survival and productivity. If so, stopover habitats in the southwestern USA may play a significant role in maintaining western populations [49], [50]. These habitats, limited and threatened by development [50], may need to be prioritized for conservation.

## Supporting Information

Table S1 All climate models describing apparent annual survival of yellow warblers breeding in Revelstoke, British Columbia (n = 279 individuals, 460 encounters). Models are ranked using QAICc. Model number refers to regionally specific climate variables described in the text. Age was included in all models as a covariate (*see* Methods). The number of parameters in the model (K), Akaike's information criterion (QAIC*_c_*), QAIC*_c_* difference from the top model (ΔQAIC*_c_*), and Akaike weight (ω_i_) are reported.
(DOCX)

Table S2 Ranked summary of AICc support for all candidate climate models and a null model (‘Year + Age’) describing (A) male arrival date (n = 210) and (B) female clutch initiation dates (n = 177) for yellow warblers in Revelstoke, British Columbia. Age (young = 1 yr; older ≥2 yrs) was included in all models as a covariate (*see* Methods). Model adjusted r^2^, the number of parameters in the model (K), Akaike's information criterion adjusted for small sample size (AIC*_c_*), AIC*_c_* difference from the top model (ΔAIC*_c_*), and Akaike weight (**ω_i_**) are reported.
(DOCX)

Table S3 Correlation (r) matrix of explanatory climate variables and time (year) (n = 8). Significant relationships (Spearman's ρ) are starred (P<0.05 = *, P<0.001 = **).
(DOCX)

Table S4 De-trended models describing apparent annual survival of yellow warblers breeding in Revelstoke, British Columbia (n = 279 individuals, 460 encounters). Models are ranked using QAICc. Model number refers to regionally specific climate variables described in the text. De-trended variables, entered in the model with the trend labeled as “(Year)”, appear in italics. Age was included in all models as a covariate (*see* Methods). The number of parameters in the model (K), Akaike's information criterion (QAIC*_c_*), QAIC*_c_* difference from the top model (ΔQAIC*_c_*), and Akaike weight (ω_i_) are reported.
(DOCX)

Table S5 Ranked summary of AICc support for de-trended climate models and the null model (‘Year + Age’) predicting (A) male arrival (n = 210) and (B) female clutch initiation dates (n = 177) for yellow warblers in Revelstoke, British Columbia. De-trended variables, entered in the model with the trend labeled as “(Year)”, appear in italics. Age (young = 1 yr; older ≥2 yrs) was included in all models as a covariate (*see* Methods). Model adjusted r^2^, the number of parameters in the model (K), Akaike's information criterion adjusted for small sample size (AIC*_c_*), AIC*_c_* difference from the top model (ΔAIC*_c_*), and Akaike weight (**ω_i_**) are reported.
(DOCX)
