# Supplementary material for: Wind Speed during Migration Influences the Survival, Timing of Breeding, and Productivity of a Neotropical Migrant, Setophaga petechia
Source: PLoS One. 2014 May 14;9(5):e97152. doi: 10.1371/journal.pone.0097152 (PMC4020938; doi:10.1371/journal.pone.0097152)
Supplement: Table S1 — All climate models describing apparent annual survival of yellow warblers breeding in Revelstoke, British Columbia (n = 279 individuals, 460 encounters). Models are ranked using QAICc. Model number refers to regionally specific climate variables described in the text. Age was included in all models as a covariate (see Methods). The number of parameters in the model (K), Akaike's information criterion (QAICc), QAICc difference from the top model (ΔQAICc), and Akaike weight (ωi) are reported. (DOCX) [file pone.0097152.s001.docx]

**Table S1.** All climate models describing apparent annual survival of yellow warblers breeding in Revelstoke, British Columbia (n=279 individuals, 460 encounters).

| **Period** | **Model #** | **Variables** | **K** | **QAICc** | **∆QAICc** | **ω_i_** |
| --- | --- | --- | --- | --- | --- | --- |
| Migration | 3a | U-WIND + AGE | 5 | 613.61 | 0 | 0.197 |
| Migration | 5a | U-WIND+V-WIND + AGE | 6 | 614.28 | 0.67 | 0.141 |
| Migration | 3b | U-WIND + AGE + SEX | 6 | 615.08 | 1.47 | 0.094 |
| Migration | 5e | U-WIND + V-WIND + AGE + SEX + U-WIND*SEX+ V-WIND*SEX | 9 | 615.33 | 1.72 | 0.083 |
| Migration | 3c | U-WIND + AGE + U-WIND*AGE | 6 | 615.52 | 1.91 | 0.076 |
| Migration | 5b | U-WIND+V-WIND + AGE + SEX | 7 | 615.82 | 2.21 | 0.065 |
| Winter | 1a | SOI_MAY-AUG_ + AGE | 5 | 616.43 | 2.82 | 0.048 |
| Migration | 3e | U-WIND + AGE + SEX + U-WIND*SEX | 7 | 616.93 | 3.32 | 0.037 |
| - | - | YEAR(nominal) + AGE | 11 | 616.94 | 3.33 | 0.037 |
| Migration | 3d | U-WIND + AGE + SEX + U-WIND*AGE | 7 | 617.05 | 3.44 | 0.035 |
| Winter | 1b | SOI_MAY-AUG_ + AGE + SEX | 6 | 618.00 | 4.39 | 0.022 |
| Migration | 5d | U-WIND + V-WIND + AGE + SEX + U-WIND*AGE + V-WIND*AGE | 9 | 618.26 | 4.65 | 0.019 |
| Winter | 1c | SOI_MAY-AUG_ + AGE + SOI_MAY-AUG_ *AGE | 6 | 618.39 | 4.78 | 0.018 |
| - | - | YEAR(nominal) + AGE + SEX | 12 | 618.60 | 4.99 | 0.016 |
| Migration | 3f | U-WIND + AGE + SEX + U-WIND*AGE + U-WIND*SEX | 8 | 618.87 | 5.26 | 0.014 |
| Migration | 5f | U-WIND + V-WIND + AGE + SEX + U-WIND*AGE + U-WIND*SEX + V-WIND*AGE + V-WIND*SEX | 11 | 619.44 | 5.84 | 0.011 |
| Breeding | 7a | MAY**°**C + AGE | 5 | 619.67 | 6.06 | 0.010 |
| Winter | 2a | SOI_DEC-MAR_ + AGE | 5 | 619.73 | 6.12 | 0.009 |
| Migration | 5d | U-WIND + V-WIND + AGE + SEX + U-WIND*AGE + V-WIND*AGE | 9 | 619.87 | 6.26 | 0.009 |
| Winter | 1d | SOI_MAY-AUG_ + AGE + SEX + SOI_MAY-AUG_ *AGE | 7 | 619.97 | 6.36 | 0.008 |
| Winter | 1e | SOI_MAY-AUG_ + AGE + SEX + SOI_MAY-AUG_ *SEX | 7 | 620.03 | 6.42 | 0.008 |
| Breeding | 7c | MAY**°**C + AGE + MAY**°**C*AGE | 6 | 620.83 | 7.22 | 0.005 |
| Winter | 2b | SOI_DEC-MAR_ + AGE + SEX | 6 | 623.48 | 7.58 | 0.004 |
| Breeding | 7b | MAY**°**C + AGE + SEX | 6 | 621.21 | 7.60 | 0.004 |
| Winter | 2c | SOI_DEC-MAR_ + AGE + SOI_DEC-MAR_*AGE | 6 | 621.69 | 8.08 | 0.003 |
| Winter | 1f | SOI_MAY-AUG_ + AGE + SEX + SOI_MAY-AUG_ *AGE + SOI_MAY-AUG_ *SEX | 8 | 622.00 | 8.39 | 0.003 |
| Breeding | 7d | MAY**°**C + AGE + SEX + MAY**°**C*AGE | 7 | 622.32 | 8.71 | 0.003 |
| Migration | 6a | MIGRATION RAIN + AGE | 5 | 622.68 | 9.07 | 0.002 |
| Winter | 2d | SOI_DEC-MAR_ + AGE + SEX + SOI_DEC-MAR_*AGE | 7 | 623.10 | 9.50 | 0.002 |
| Winter | 2e | SOI_DEC-MAR_ + AGE + SEX + SOI_DEC-MAR_*SEX | 7 | 623.19 | 9.59 | 0.002 |
| Breeding | 7e | MAY**°**C + AGE + SEX + MAY**°**C*SEX | 7 | 623.27 | 9.66 | 0.002 |
| Migration | 4a | V-WIND + AGE | 5 | 623.50 | 9.89 | 0.001 |
| Migration | 4e | V-WIND + AGE + SEX + V *SEX | 7 | 623.53 | 9.92 | 0.001 |
| Migration | 6b | MIGRATION RAIN + AGE + SEX | 6 | 624.24 | 10.63 | 0.001 |
| Breeding | 7f | MAY**°**C + AGE + SEX + MAY**°**C*AGE + MAY**°**C*SEX | 8 | 624.36 | 10.75 | 0.001 |
| Migration | 6c | MIGRATION RAIN + AGE + MIG RAIN*AGE | 6 | 624.50 | 10.89 | 0.001 |
| Migration | 6e | MIGRATION RAIN + AGE + SEX + MIG RAIN*SEX | 7 | 624.83 | 11.22 | 0.001 |
| Migration | 4b | V-WIND + AGE + SEX | 6 | 625.01 | 11.40 | 0.001 |
| Winter | 2f | SOI_DEC-MAR_ + AGE + SEX + SOI_DEC-MAR_*AGE + SOI_DEC-MAR_*SEX | 7 | 625.15 | 11.55 | 0.001 |
| Migration | 6d | MIGRATION RAIN + AGE + SEX + MIG RAIN*AGE | 7 | 625.31 | 11.70 | 0.001 |
| Migration | 4c | V-WIND + AGE + V *AGE | 6 | 625.49 | 11.88 | 0.001 |
| Migration | 4f | V-WIND + AGE + SEX + V-WIND*AGE + V-WIND*SEX | 8 | 625.59 | 11.98 | 0.001 |
| Migration | 6f | MIGRATION RAIN + AGE + SEX + MIG RAIN*AGE + MIG RAIN*SEX | 8 | 626.29 | 12.68 | 0 |
| Migration | 4e | V-WIND + AGE + SEX + V-WIND*AGE | 7 | 627.04 | 13.43 | 0 |
| - | - | YEAR(nominal)*AGE*SEX | 34 | 643.45 | 29.84 | 0 |
