# Supplementary material for: Wind Speed during Migration Influences the Survival, Timing of Breeding, and Productivity of a Neotropical Migrant, Setophaga petechia
Source: PLoS One. 2014 May 14;9(5):e97152. doi: 10.1371/journal.pone.0097152 (PMC4020938; doi:10.1371/journal.pone.0097152)
Supplement: Table S2 — Ranked summary of AICc support for all candidate climate models and a null model (‘Year + Age’) describing (A) male arrival date (n = 210) and (B) female clutch initiation dates (n = 177) for yellow warblers in Revelstoke, British Columbia. Age (young = 1 yr; older ≥2 yrs) was included in all models as a covariate (see Methods). Model adjusted r2, the number of parameters in the model (K), Akaike's information criterion adjusted for small sample size (AICc), AICc difference from the top model (ΔAICc), and Akaike weight (ωi) are reported. (DOCX) [file pone.0097152.s002.docx]

**Table S2**. Ranked summary of AICc support for all candidate climate models and a null model (YEAR + AGE) describing (A) male arrival date (n=210) and (B) female clutch initiation dates (n=177) for yellow warblers in Revelstoke, British Columbia.

| **A) Male Arrival** | | | | | |  |  |
| --- | --- | --- | --- | --- | --- | --- | --- |
| **Period** | **Model #** | **Variables** | **r^2^** | **K** | **AIC.c** | **∆AICc** | **ω_i_** |
| Migration | 3b | U-WIND + AGE + U-WIND*AGE | 0.21 | 5 | 1452.88 | 0 | 0.535 |
| Winter | 1b | SOI_MAY-AUG_ + AGE +SOI_MAY-AUG_ *AGE | 0.20 | 5 | 1453.85 | 0.98 | 0.328 |
| Migration | 5b | U-WIND + V-WIND + AGE + V-WIND*AGE + U-WIND*AGE | 0.20 | 7 | 1456.14 | 3.27 | 0.105 |
| Migration | 3a | U-WIND + AGE | 0.18 | 4 | 1460.30 | 7.42 | 0.013 |
| Winter | 1a | SOI_MAY-AUG_ + AGE | 0.17 | 4 | 1461.16 | 8.29 | 0.008 |
| Migration | 5a | U-WIND + V-WIND + AGE | 0.17 | 5 | 1462.35 | 9.47 | 0.005 |
| Winter | 2b | SOI_DEC-MAR_ + SOI_DEC-MAR_ *AGE | 0.17 | 5 | 1463.02 | 10.15 | 0.003 |
| - | - | YEAR+AGE | 0.18 | 9 | 1464.32 | 11.44 | 0.002 |
| Winter | 2a | SOI_DEC-MAR_ + AGE | 0.15 | 4 | 1465.49 | 12.62 | 0.001 |
| Breeding | 7b | MAY**°**C + AGE + MAY**°**C *AGE | 0.11 | 5 | 1477.64 | 24.76 | 0 |
| Migration | 4a | V-WIND + AGE | 0.10 | 4 | 1478.30 | 25.42 | 0 |
| Breeding | 7a | MAY**°**C + AGE | 0.10 | 4 | 1478.34 | 25.46 | 0 |
| Migration | 4b | V-WIND + AGE + V-WIND*AGE | 0.10 | 5 | 1480.09 | 27.21 | 0 |
| Migration | 6a | MIGRATION RAIN + AGE | 0.08 | 4 | 1482.70 | 29.82 | 0 |
| Migration | 6b | MIGRATION RAIN + MIG RAIN*AGE | 0.08 | 5 | 1484.75 | 31.88 | 0 |
| **B) Female clutch initiation date** | | | | | | | |
| **Period** | **Model #** | **Variables** | **r^2^** | **K** | **AIC.c** | **∆AICc** | **ω_i_** |
| Migration | 3b | U-WIND + AGE + U-WIND*AGE | 0.16 | 5 | 1194.89 | 0 | 0.289 |
| Migration | 5a | U-WIND + V-WIND + AGE | 0.15 | 5 | 1195.00 | 0.11 | 0.273 |
| Migration | 3a | U-WIND + AGE | 0.14 | 4 | 1195.82 | 0.93 | 0.181 |
| Migration | 5b | U-WIND + V-WIND + AGE + V-WIND*AGE + U-WIND*AGE | 0.16 | 7 | 1196.47 | 1.58 | 0.131 |
| - | - | YEAR + AGE | 0.16 | 9 | 1197.90 | 3.00 | 0.064 |
| Winter | 1a | SOI_MAY-AUG_ + AGE | 0.12 | 4 | 1200.00 | 5.09 | 0.023 |
| Winter | 1b | SOI_MAY-AUG_ + AGE +SOI_MAY-AUG_ *AGE | 0.12 | 5 | 1201.54 | 6.65 | 0.01 |
| Winter | 2b | SOI_DEC-MAR_ + SOI_DEC-MAR_ *AGE | 0.12 | 5 | 1202.50 | 7.61 | 0.006 |
| Breeding | 7a | MAY**°**C + AGE | 0.11 | 4 | 1202.76 | 7.87 | 0.006 |
| Winter | 2a | SOI_DEC-MAR_ + AGE | 0.11 | 4 | 1202.84 | 7.95 | 0.005 |
| Migration | 6a | MIGRATION RAIN +AGE | 0.11 | 4 | 1203.10 | 8.22 | 0.005 |
| Breeding | 7b | MAY**°**C + AGE + MAY**°**C*AGE | 0.11 | 5 | 1204.58 | 9.69 | 0.002 |
| Migration | 4a | V-WIND+ AGE | 0.10 | 4 | 1205.00 | 10.11 | 0.002 |
| Migration | 6b | MIGRATION RAIN + MIG RAIN*AGE | 0.10 | 5 | 1205.07 | 10.18 | 0.002 |
| Migration | 4b | V-WIND+ AGE + V-WIND*AGE | 0.10 | 5 | 1206.54 | 11.65 | 0.001 |
