# Supplementary material for: Wind Speed during Migration Influences the Survival, Timing of Breeding, and Productivity of a Neotropical Migrant, Setophaga petechia
Source: PLoS One. 2014 May 14;9(5):e97152. doi: 10.1371/journal.pone.0097152 (PMC4020938; doi:10.1371/journal.pone.0097152)
Supplement: Table S3 — Correlation (r) matrix of explanatory climate variables and time (year) (n = 8). Significant relationships (Spearman's ρ) are starred (P<0.05 = *, P<0.001 = **). (DOCX) [file pone.0097152.s003.docx]

**Table S3.** Correlation (r) matrix of explanatory climate variables and time (year) (n=8). Significant relationships (Spearman’s ρ) are starred (P<0.05=*, P<0.001=**).

|  | **May°C** | **Mig Rain_Mar-May_** | **U-Wind_Mar-May_** | **V-Wind_Mar-May_** | **SOI_May-Aug_** | **SOI_Dec-Mar_** |
| --- | --- | --- | --- | --- | --- | --- |
| **Year** | -0.94** | 0.16 | 0.74* | 0.15 | 0.72* | 0.45 |
| **May°C** |  | 0.03 | -0.60 | -0.24 | -0.60** | -0.46 |
| **Mig Rain_Mar-May_** |  |  | -0.04 | 0.49 | 0.30 | -0.29 |
| **U-Wind_Mar-May_** |  |  |  | -0.36 | 0.75* | 0.69* |
| **V-Wind_Mar-May_** |  |  |  |  | 0.18 | 0.00 |
| **SOI_May-Aug_** |  |  |  |  |  | 0.75* |
