# Supplementary material for: Wind Speed during Migration Influences the Survival, Timing of Breeding, and Productivity of a Neotropical Migrant, Setophaga petechia
Source: PLoS One. 2014 May 14;9(5):e97152. doi: 10.1371/journal.pone.0097152 (PMC4020938; doi:10.1371/journal.pone.0097152)
Supplement: Table S4 — De-trended models describing apparent annual survival of yellow warblers breeding in Revelstoke, British Columbia (n = 279 individuals, 460 encounters). Models are ranked using QAICc. Model number refers to regionally specific climate variables described in the text. De-trended variables, entered in the model with the trend labeled as “(Year)”, appear in italics. Age was included in all models as a covariate (see Methods). The number of parameters in the model (K), Akaike's information criterion (QAICc), QAICc difference from the top model (ΔQAICc), and Akaike weight (ωi) are reported. (DOCX) [file pone.0097152.s004.docx]

**Table S4.** De-trended models describing apparent annual survival of yellow warblers breeding in Revelstoke, British Columbia (n=279 individuals, 460 encounters).

| **Period** | **Model #** | **Variables** | **K** | **QAICc** | **∆QAICc** | **ω_i_** |
| --- | --- | --- | --- | --- | --- | --- |
| Migration | 3a | (Year) + *U-WIND* + AGE | 6 | 614.14 | 0 | 0.078 |
| Breeding | 7a | (Year) + *MAY****°****C* + AGE | 6 | 614.42 | 0.28 | 0.068 |
| Migration | 4e | (Year) + *V-WIND* + AGE + SEX + *V-WIND**SEX | 8 | 614.62 | 0.48 | 0.061 |
| Winter | 2a | (Year) + *SOI_DEC-MAR_* + AGE | 6 | 614.77 | 0.63 | 0.057 |
| Winter | 1a | (Year) + *SOI_MAY-AUG_* + AGE | 6 | 615.30 | 1.16 | 0.044 |
| Migration | 4a | (Year) + *V-WIND* + AGE | 6 | 615.34 | 1.20 | 0.043 |
| Migration | 6a | (Year) + *MIGRATION RAIN* + AGE | 6 | 615.43 | 1.29 | 0.041 |
| Migration | 3c | (Year) + *U-WIND* + AGE + *U-WIND**AGE | 7 | 615.57 | 1.43 | 0.038 |
| Migration | 3b | (Year) + *U-WIND* + AGE + SEX | 7 | 615.66 | 1.52 | 0.036 |
| Breeding | 7c | (Year) + *MAY****°****C* + AGE + *MAY****°****C**AGE | 7 | 615.88 | 1.74 | 0.033 |
| Migration | 5a | (Year) + *U-WIND* + *V-WIND* + AGE | 7 | 615.97 | 1.83 | 0.031 |
| Breeding | 7b | (Year) + *MAY****°****C* + AGE + SEX | 7 | 615.98 | 1.84 | 0.031 |
| Migration | 5e | (Year) + *U-WIND* + *V-WIND* + AGE + SEX + *U-WIND**SEX + *V-WIND**SEX | 10 | 616.20 | 2.06 | 0.028 |
| Migration | 6c | (Year) + *MIGRATION RAIN* + AGE + *MIG RAIN**AGE | 7 | 616.23 | 2.09 | 0.027 |
| Winter | 2b | (Year) + *SOI_DEC-MAR_*+ AGE + SEX | 7 | 616.30 | 2.16 | 0.026 |
| Migration | 4f | (Year) + *V-WIND* + AGE + SEX + *V-WIND**AGE + *V-WIND**SEX | 9 | 616.60 | 2.46 | 0.024 |
| Winter | 2c | (Year) + *SOI_DEC-MAR_*+ AGE + *SOI_DEC-MAR_**AGE | 7 | 617.49 | 2.61 | 0.021 |
| Winter | 1b | (Year) + *SOI_MAY-AUG_* + AGE + SEX | 7 | 616.87 | 2.73 | 0.020 |
| Migration | 6e | (Year) + *MIGRATION RAIN* + AGE + SEX + *MIG RAIN**SEX | 8 | 616.87 | 2.73 | 0.020 |
| Migration | 4b | (Year) + *V-WIND* + AGE + SEX | 7 | 616.89 | 2.75 | 0.020 |
| - | - | YEAR(nominal) + AGE | 11 | 616.94 | 2.80 | 0.019 |
| Migration | 6b | (Year) + *MIGRATION RAIN* + AGE + SEX | 7 | 616.98 | 2.84 | 0.019 |
| Winter | 1c | (Year) + *SOI_MAY-AUG_* + AGE + *SOI_MAY-AUG_**AGE | 7 | 617.15 | 3.01 | 0.017 |
| Migration | 3d | (Year) + *U-WIND* + AGE + SEX + *U-WIND**AGE | 8 | 617.21 | 3.07 | 0.017 |
| Migration | 3e | (Year) + *U-WIND* + AGE + SEX + *U-WIND**SEX | 8 | 617.22 | 3.08 | 0.017 |
| Migration | 4c | (Year) + *V-WIND* + AGE + *V-WIND**AGE | 7 | 617.27 | 3.13 | 0.016 |
| Breeding | 7d | (Year) + *MAY****°****C* + AGE + SEX + *MAY****°****C**AGE | 8 | 617.34 | 3.20 | 0.016 |
| Migration | 6d | (Year) + *MIGRATION RAIN* + AGE + SEX + *MIG RAIN**AGE | 8 | 617.52 | 3.38 | 0.014 |
| Migration | 5b | (Year) + *U-WIND* + *V-WIND* + AGE + SEX | 8 | 617.52 | 3.38 | 0.014 |
| Migration | 6f | (Year) + *MIGRATION RAIN* + AGE + SEX + *MIG RAIN**AGE + *MIG RAIN**SEX | 9 | 617.84 | 3.70 | 0.012 |
| Breeding | 7e | (Year) + *MAY****°****C* + AGE + SEX + *MAY****°****C**SEX | 8 | 618.03 | 3.89 | 0.011 |
| Winter | 2d | (Year) + *SOI_DEC-MAR_* + AGE + SEX + *SOI_DEC-MAR_* *AGE | 8 | 618.24 | 4.10 | 0.010 |
| Winter | 2e | (Year) + *SOI_DEC-MAR_* + AGE + SEX + *SOI_DEC-MAR_* *SEX | 8 | 618.35 | 4.21 | 0.010 |
| - | - | YEAR(nominal) + AGE + SEX | 12 | 618.60 | 4.46 | 0.008 |
| Winter | 1d | (Year) + *SOI_MAY-AUG_* + AGE + SEX + *SOI_MAY-AUG_**AGE | 8 | 618.72 | 4.58 | 0.008 |
| Migration | 3f | (Year) + *U-WIND* + AGE + SEX + *U-WIND**AGE + *U-WIND**SEX | 9 | 618.76 | 4.62 | 0.008 |
| Migration | 4d | (Year) + *V-WIND* + AGE + SEX + *V-WIND**AGE | 8 | 618.87 | 4.73 | 0.007 |
| Winter | 1e | (Year) + *SOI_MAY-AUG_* + AGE + SEX + *SOI_MAY-AUG_**SEX | 8 | 618.90 | 4.76 | 0.007 |
| Breeding | 7f | (Year) + *MAY****°****C* + AGE + SEX + *MAY****°****C**AGE + *MAY****°****C**SEX | 9 | 619.30 | 5.16 | 0.006 |
| Migration | 5c | (Year) + *U-WIND* + *V-WIND* + AGE + *U-WIND**AGE + *V-WIND**AGE | 9 | 619.43 | 5.29 | 0.006 |
| Migration | 5f | (Year) + *U-WIND* + *V-WIND* + AGE + SEX + *U-WIND**AGE + *V-WIND**AGE+ *U-WIND**SEX + *V-WIND**SEX | 12 | 620.12 | 5.98 | 0.004 |
| Winter | 2f | (Year) + *SOI_DEC-MAR_* + AGE + SEX + *SOI_DEC-MAR_* *AGE + *SOI_DEC-MAR_* *SEX | 9 | 620.31 | 6.17 | 0.004 |
| Winter | 1f | (Year) + *SOI_MAY-AUG_* + AGE + SEX + *SOI_MAY-AUG_**AGE + *SOI_MAY-AUG_**SEX | 9 | 620.73 | 6.59 | 0.003 |
| Migration | 5d | (Year) + *U-WIND* + *V-WIND* + AGE + SEX + *U-WIND**AGE + *V-WIND**AGE | 10 | 621.10 | 6.96 | 0.002 |
