# Supplementary material for: Wind Speed during Migration Influences the Survival, Timing of Breeding, and Productivity of a Neotropical Migrant, Setophaga petechia
Source: PLoS One. 2014 May 14;9(5):e97152. doi: 10.1371/journal.pone.0097152 (PMC4020938; doi:10.1371/journal.pone.0097152)
Supplement: Table S5 — Ranked summary of AICc support for de-trended climate models and the null model (‘Year + Age’) predicting (A) male arrival (n = 210) and (B) female clutch initiation dates (n = 177) for yellow warblers in Revelstoke, British Columbia. De-trended variables, entered in the model with the trend labeled as “(Year)”, appear in italics. Age (young = 1 yr; older ≥2 yrs) was included in all models as a covariate (see Methods). Model adjusted r2, the number of parameters in the model (K), Akaike's information criterion adjusted for small sample size (AICc), AICc difference from the top model (ΔAICc), and Akaike weight (ωi) are reported. (DOCX) [file pone.0097152.s005.docx]

**Table S5.** Ranked summary of AICc support for de-trended climate models and the null model (YEAR + AGE) predicting (A) male arrival (n=210) and (B) female clutch initiation dates (n=177) for yellow warblers in Revelstoke, British Columbia.

| **A) Male Arrival** | | | | | | | |
| --- | --- | --- | --- | --- | --- | --- | --- |
| **Period** | **Model #** | **Variables** | **r^2^** | **K** | **AIC.c** | **∆AICc** | **ω_i_** |
| Migration | 3b | (Year) + *U-WIND* + AGE + *U-WIND**AGE | 0.18 | 6 | 1460.48 | 0 | 0.357 |
| Migration | 3a | (Year) + *U-WIND* + AGE | 0.17 | 5 | 1461.95 | 1.47 | 0.171 |
| Winter | 1a | (Year) + *SOI_MAY-AUG_* + AGE | 0.17 | 5 | 1462.75 | 2.28 | 0.114 |
| Winter | 1b | (Year) + *SOI_MAY-AUG_* + AGE + *SOI_MAY-AUG_**AGE | 0.17 | 6 | 1462.89 | 2.41 | 0.107 |
| Migration | 5a | (Year) + *U-WIND* + *V-WIND* + AGE | 0.17 | 6 | 1463.19 | 2.72 | 0.092 |
| - | - | YEAR (nominal) + AGE | 0.18 | 9 | 1464.32 | 3.84 | 0.052 |
| Migration | 5b | (Year) + *U-WIND* + *V-WIND* + AGE + *V-WIND**AGE + *U-WIND**AGE | 0.18 | 8 | 1464.36 | 3.88 | 0.051 |
| Winter | 2a | (Year) + *SOI_DEC-MAR_* + AGE | 0.16 | 5 | 1465.32 | 4.84 | 0.032 |
| Winter | 2b | (Year) + *SOI_DEC-MAR_* + *SOI _DEC-MAR_**AGE | 0.16 | 6 | 1466.16 | 5.68 | 0.021 |
| Migration | 4a | (Year) + *V-WIND* + AGE | 0.13 | 5 | 1471.33 | 10.85 | 0.002 |
| Migration | 4b | (Year) + *V-WIND* + AGE + *V-WIND**AGE | 0.14 | 6 | 1472.05 | 11.57 | 0.001 |
| Breeding | 7b | (Year) + *MAY****°****C* + AGE + *MAY****°****C**AGE | 0.12 | 6 | 1475.59 | 15.12 | 0 |
| Breeding | 7a | (Year) + *MAY****°****C* + AGE | 0.12 | 5 | 1475.61 | 15.13 | 0 |
| Migration | 6a | (Year) + *MIGRATION RAIN* + AGE | 0.12 | 5 | 1476.05 | 15.57 | 0 |
| Migration | 6b | (Year) + *MIGRATION RAIN* + *MIG RAIN**AGE | 0.11 | 6 | 1478.17 | 17.69 | 0 |
| **B) Female Clutch Initiation Date** | | | | | | | |
| **Period** | **Model #** | **Variables** | **r^2^** | **K** | **AIC.c** | **∆AICc** | **ω_i_** |
| Migration | 5a | (Year) + *U-WIND* + *V-WIND* + AGE | 0.15 | 6 | 1196.13 | 0 | 0.243 |
| Migration | 3b | (Year) + *U-WIND* + AGE + *U-WIND**AGE | 0.15 | 6 | 1197.04 | 0.91 | 0.154 |
| Migration | 3a | (Year) + *U-WIND* + AGE | 0.14 | 5 | 1197.75 | 1.63 | 0.108 |
| Breeding | 7a | (Year) + *MAY****°****C* + AGE | 0.14 | 5 | 1197.76 | 1.64 | 0.107 |
| - | - | YEAR (nominal) + AGE | 0.16 | 9 | 1197.90 | 1.769 | 0.100 |
| Migration | 5b | (Year) + *U-WIND* + *V-WIND* + AGE + *V-WIND**AGE + *U-WIND**AGE | 0.16 | 8 | 1197.96 | 1.829 | 0.097 |
| Breeding | 7b | (Year) + *MAY****°****C* + AGE + *MAY****°****C**AGE | 0.14 | 6 | 1199.42 | 3.297 | 0.047 |
| Migration | 6a | (Year) + *MIGRATION RAIN* + AGE | 0.13 | 5 | 1200.20 | 4.069 | 0.032 |
| Winter | 1a | (Year) + *SOI_MAY-AUG_* + AGE | 0.13 | 5 | 1200.84 | 4.718 | 0.023 |
| Winter | 2a | (Year) + *SOI_DEC-MAR_* + AGE | 0.13 | 5 | 1200.87 | 4.74 | 0.023 |
| Winter | 2b | (Year) + *SOI_DEC-MAR_* + *SOI _DEC-MAR_**AGE | 0.13 | 6 | 1201.32 | 5.191 | 0.018 |
| Migration | 4a | (Year) + *V-WIND* + AGE | 0.12 | 5 | 1201.33 | 5.204 | 0.018 |
| Migration | 6b | (Year) + *MIGRATION RAIN* + *MIG RAIN**AGE | 0.13 | 6 | 1201.88 | 5.757 | 0.014 |
| Migration | 4b | (Year) + *V-WIND* + AGE + *V-WIND**AGE | 0.12 | 6 | 1202.55 | 6.426 | 0.010 |
| Winter | 1b | (Year) + *SOI_MAY-AUG_* + AGE + *SOI_MAY-AUG_**AGE | 0.12 | 6 | 1202.85 | 6.725 | 0.008 |
